# MarA binds and activates the ABC efflux pump YadGH to confer conditional macrolide resistance in AcrB-deficient *Escherichia coli*

**DOI:** 10.3389/fmicb.2026.1902331

**Published:** 2026-07-29

**Authors:** Zhenyue Feng, Jiqin Li, Biao Ma, Hong Wang, Yanli Li, Liangrong Bao, Defu Liu

**Affiliations:** 1 College of Agronomy and Life Sciences, Zhaotong University, Zhaotong, China; 2 Yunnan Engineering Research Center of Green Planting and Processing of Gastrodia, Zhaotong University, Zhaotong, China; 3 Institute for Advanced Study on Ecological Agriculture and Biodiversity of the Yunnan-Guizhou Plateau, Zhaotong University, Zhaotong, China; 4 Yunnan Key Laboratory of Smart Villages and Agri-Cultural-Tourism Integration, Zhaotong University, Zhaotong, China

**Keywords:** ABC efflux pump, macrolides, MarA, transcriptional regulation, YadGH

## Abstract

**Background:**

The global transcriptional regulator MarA drives multidrug resistance in *Escherichia coli*. MarA regulation of Resistance-Nodulation-Cell Division (RND) efflux pumps such as AcrAB-TolC is well established, but its control over ATP-binding cassette (ABC)-type efflux pumps remains unclear. The ABC efflux pump YadGH exports macrolides, yet its upstream transcriptional regulation is unknown.

**Methods:**

Deletion mutants Δ*marA*, Δ*yadG*, Δ*yadH*, and double mutants were constructed in *E. coli* K-12 MG1655. Minimum inhibitory concentrations (MICs) of 15 antibiotics were measured by broth microdilution. *YadGH* transcription was assessed by qRT-PCR. MarA binding to the *yadGH* promoter was verified by electrophoretic mobility shift assay (EMSA) and chromatin immunoprecipitation quantitative real-time PCR (ChIP-qPCR). Biofilm formation and swarming motility were assayed.

**Results:**

MIC assays revealed that single deletions of *yadG* or *yadH* had negligible effects on most antibiotics. However, in the Δ*acrB* background, deleting *yadGH* (Δ*acrB*Δ*yadG* and Δ*acrB*Δ*yadH*) increased sensitivity to erythromycin and tylosin, indicating that YadGH conditionally contributes to macrolide resistance specifically when the major efflux pump AcrB is absent. qRT-PCR showed that *yadGH* transcription is induced in a MarA-dependent manner in *E. coli* K-12. The Δ*marA* mutant phenocopied this susceptibility profile, supporting the role of *yadGH* as a downstream effector of MarA within this genetic context. ChIP-qPCR confirmed MarA binding to the *yadGH* promoter, and EMSA mapped a degenerate marbox motif upstream of the *yadG* start codon. Genome annotation lists *yadG* and *yadH* as a two-gene operon.

**Conclusion:**

MarA binds to the *yadGH* promoter and positively regulates its transcription. YadGH serves as a conditional auxiliary efflux pump for macrolides when AcrAB-TolC is absent. Deletion of *yadGH* also affects biofilm formation and motility.

## 1 Introduction

The long-term, intensive use of veterinary macrolides such as tylosin in livestock farming leaves environmental drug residues that impose selective pressure on *E. coli* (Shen et al., 2025). This pressure drives the evolution of multiple defense mechanisms (Ma et al., 2022), including active efflux, drug inactivation, target modification, reduced outer membrane permeability, and biofilm formation (Ma et al., 2022; Elshobary et al., 2025). Among these mechanisms, overexpression of efflux pumps is a major driver of bacterial resistance. Studies have shown that in prokaryotes, seven superfamilies of multidrug efflux pumps can confer antibiotic resistance (Nasrollahian et al., 2024). Notably, although some of these pumps only confer low-level resistance, they often mark the first step in resistance development and can eventually lead to higher-level resistance through acquired chromosomal mutations targeting antibiotics (Chitsaz and Brown, 2017; Frimodt-Møller et al., 2018). The ABC superfamily is one such group of efflux pumps (Huda et al., 2003). As one of the largest protein superfamilies, ABC transporters harness the energy released from ATP hydrolysis to mediate the transport of structurally diverse molecules (Orelle et al., 2019; Nasrollahian et al., 2024); their substrates range from antibiotics and virulence factors to signaling molecules (Greene et al., 2018). Since *E. coli* is a classic model organism whose genome was sequenced as early as 1997 (Blattner et al., 1997), researchers have systematically characterized the ABC family repertoire (Moussatova et al., 2008), representing its largest protein family. *E. coli* encodes about 80 ABC family proteins. These assemble into 72 independent functional systems (as some systems comprise multiple subunits), of which 11 are considered efflux proteins, and 7 have been confirmed or are predicted to act as drug efflux pumps (Moussatova et al., 2008). Although bioinformatics predicts that YadGH has efflux capability, direct experimental evidence clarifying its exact contribution to antibiotic resistance and its regulatory mechanisms remains lacking.

YadGH is composed of the cytoplasmic ATPase subunit YadG, which contains a nucleotide-binding domain and conserved functional sequences, and the membrane-integrated subunit YadH, which has six-transmembrane domains. Efflux systems in Gram-negative bacteria frequently feature functional redundancy. The primary transporter AcrAB-TolC carries out most antimicrobial clearance under normal growth conditions. As a tentative working model, smaller auxiliary pumps such as YadGH are proposed to serve as compensatory secondary machineries. These transporters are only robustly upregulated once AcrAB-TolC is deleted or functionally disrupted. This potential compensatory mechanism could restore partial drug efflux capacity and help bacteria reduce metabolic energy costs when no stress is present (Sulavik et al., 2001; Nikaido, 2009). However, precisely because of this phenotypic concealment, the upstream transcriptional regulation of auxiliary efflux pumps, especially under the clinically and experimentally common background of AcrAB-TolC deletion-has long remained unclear, leaving a gap in our understanding of how bacteria activate auxiliary pumps upon failure of the major pump.

Transcriptional regulation of efflux pumps is critical for bacterial adaptation to external stress (Du et al., 2018). The global transcription factor MarA belongs to the AraC family, is encoded in the *marRAB* operon, and is a central regulator of the multidrug resistance network in *E. coli* (Alekshun and Levy, 1999; Barbosa and Levy, 2000). MarA recognizes the marbox motif (5′-TGTAT-W-T-W-AA-3′) in promoter regions (Alekshun and Levy, 1997; Trigg et al., 2025) and regulates the transcription of hundreds of functional genes, including efflux pumps, metabolic enzymes, and virulence-related genes. In contrast, the global silencer protein H-NS often binds to AT-rich promoters and represses gene transcription through oligomerization (Dorman, 2004; Liu et al., 2024). At certain loci, MarA can relieve this silencing by binding to its target sequences, establishing an antagonistic relationship with H-NS (Dorman, 2004). Whether such antagonism occurs at the yadGH promoter, however, remains unknown and is one of the hypotheses examined in this study. Although the MarA regulon was recently expanded to include genes involved in DNA repair (*xseA*) and outer membrane lipid asymmetry (*mlaFEDCB*) (Sharma et al., 2017), direct MarA targeting of the *yadGH* operon has not been reported. Early transcriptomic studies suggested that *yadG* and *yadH* are potential MarA targets (Barbosa and Levy, 2000), but direct DNA binding and the potential interplay with nucleoid-associated proteins such as H-NS were not examined. Whether MarA regulates the *yadGH* operon, particularly in the context of AcrAB-TolC deficiency, remains unresolved.

Beyond antibiotic efflux, some ABC family transporters have also been reported to participate in the regulation of physiological processes such as bacterial biofilm formation and motility (Benda et al., 2021; How et al., 2024). Consistent with this, in our preliminary experiments, we observed that the *yadGH* knockout strain not only showed increased susceptibility to tylosin, but also exhibited changes in biofilm formation and swarming motility, suggesting that YadGH may have multiple physiological functions.

Based on these observations, we propose the following working hypothesis: MarA may activate *yadGH* transcription by counteracting H-NS-dependent silencing at its promoter. In this study, we used *E. coli* K-12 MG1655 as a model and employed gene knockout, transcriptional analysis, EMSA, and ChIP-qPCR to systematically dissect the transcriptional regulatory mechanism by which MarA controls *yadGH*. In this study, we use *E. coli* K-12 MG1655 as a model and employ gene knockout, transcriptional analysis, DNA pull-down, EMSA, and ChIP-qPCR to systematically dissect the transcriptional regulatory mechanism by which MarA controls *yadGH*. Furthermore, future work will examine whether YadGH can compensate for impaired efflux capacity via MarA-dependent upregulation and confer macrolide resistance when AcrAB-TolC is non-functional, to evaluate its putative function as a secondary compensatory efflux system.

## 2 Materials and methods

### 2.1 Bacterial strains, plasmids, and primers

All bacterial strains and plasmids generated and employed in this work are summarized in Table 1. The wild-type *E. coli* K-12 MG1655 served as the parental strain for all mutant derivatives. Gene knockout mutants were constructed using the suicide vector pDM4 or the λ-Red recombination system. For heterologous protein expression, the *marA* gene was cloned into pET-32a(+), and the construct was transformed into *E. coli* BL21(DE3). All synthetic oligonucleotides and primers used in this work are listed in Table 2.

**TABLE 1:** Bacterial strains used in this study.

Bacterial strains and plasmid	Genotype/description	Reference or source
Bacterial strains		
*E. coli* K-12 MG1655	Wild type	Lab collection
*S17-1 λpir*	Conjugation donor	Lab collection
*E. coli*χ*7213*	ΔdapA::erm, *λpir*, Sm_*r*_, *recA*	Lab collection
*E. coli*/pET32a	pET expression vector	Lab collection
*E. coli* Δ*marA*	*marA* deletion mutant	This study
*E. coli* Δ*yadG*	*yadG* deletion mutant	This study
*E. coli* Δ*yadH*	*yadH* deletion mutant	This study
*E. coli* Δ*acrB*	*acrB* deletion mutant	This study
*E. coli* Δ*acrB*Δ*yadG*	Double deletion mutant	This study
*E. coli* Δ*acrB*Δ*yadH*	Double deletion mutant	Gift from Prof. Cui
*E. coli*/pET28a-*yadG*	pET 28a- *ybhG*, Kan ^r^	This study
*E. coli*/pET32a-*marA*	pET 32a- *marA*, Kan ^r^	This study
pGBKT7-*marA*	TRP1, 2μ origin, GAL4 DNA-binding domain (BD) fusion	This study
pGADT7-*yadG*	Amp_*r*_, Leu^–^, GAL4 AD fusion vector; expresses YadG-AD	This study
Plasmids		
pET32a	Over-expression vector	Lab collection
pKD46	bla (Ap ^r^), a helper plasmid for expression of Redλ recombinase	Lab collection
pKD3	Template plasmid for a chloramphenicol resistance gene	Lab collection
PCP20	bla (Ap ^r^) cat (Cm ^r^), a plasmid for expression of FLP recombinase	Lab collection
pGBKT7	GAL4 BD fusion vector, Trp^−^ selection	Realgene Bioscience, Nanjing, China
pGADT7	GAL4 AD fusion vector, Leu^−^ selection	Realgene Bioscience, Nanjing, China
pDM4	Suicide vector, R6K ori (λpir^+^ host only), Cm*^r^*, *sacB*	Lab collection

**TABLE 2:** Primers used in this study.

Primer name	Sequence (5′–3′)	Size (bp)	Application
*marA*-χ7213-F	CTGAAGATCAGCAGTTCAACCTGT	24	Identification of transformed χ7213 strain
*marA*-χ7213-R	TGTTCAGCTACTGACGGGGTG	21	Identification of transformed χ7213 strain
*marA*-3	GGACCTGCACCAAGAATT	18	Knockout of *marA*
*marA*-4	GGTAGAGCGTCGTAGGGA	18	Knockout of *marA*
*marA*-F	CGCGGATCCATGTCCAGACGCAATACTGACG	31	Overexpression vector construction
*marA*-R	CCCAAGCTTGCTGTTGTAATGATTTAATGGATGTAA	36	Overexpression vector construction
pDK46-F	GACAAAGGCACCACCATC	18	pKD46 plasmid validation
pDK46-R	CTAACATCGCCTTCCTCC	18	pKD46 plasmid validation
T-*yadG*-F	GCCAGTTGTTGATAAGCC	18	Knockout of *yadG*
T-*yadG*-R	GTCACCCAGAATGCCAACA	19	Knockout of *yadG*
*Cm*-F	TGAAACTCACCCAGGGATTG	20	Validation of chloramphenicol resistance gene
*Cm*-R	ATAAATCCTGGTGTCCCTGT	20	Validation of chloramphenicol resistance gene
K-*yadH*-F	ATGATGCATCTTTACTGGTGGCCTA AAAAGCATCTGGCGAAATTGAG	47	*yadH* knockout targeting fragment
K-*yadH*-R	CAGACTAGCTAGAACCGAGGCACGTT GATGCAGCGACAACAGATTAACGG	50	*yadH* knockout targeting fragment
T-*yadH*-F	GAGAACAGGGCCACATGC	18	Knockout of *yadH*
T-*yadH*-R	CTAAACTCACCTCCGCC	17	Knockout of *yadH*
WT-probe-bio-F	CGCGAGCCTGTATTGTTAATTGCCATAAAGAG	32	Preparation of biotin-labeled wild-type probe for EMSA
WT-probe-bio-R	CTCTTTATGGCAATTAACAATACAGGCTCGCG	32	Preparation of biotin-labeled wild-type probe for EMSA
Mut-probe-bio-F	CGCGAGCATTGATTGTTAATTGCCATAAAGAG	32	Preparation of biotin-labeled mutant probe for EMSA
Mut-probe-bio-R	CTCTTTATGTCCATGCAAACTCAATGCTCGCG	32	Preparation of biotin-labeled mutant probe for EMSA
*yadG*-F1	GCGGGTGATTTTTATGCGCT	20	Amplification of *yadG* promoter for ChIP-qPCR
*yadG*-R1	GCGTTCACGACATCCTTCTC	20	Amplification of *yadG* promoter for ChIP-qPCR
GapA-F:	GCTGGTGCGAAGAAAGTGG	19	Amplification of qPCR reference gene
GapA-R:	GGTAGTAGCGTGAACGGTGG	20	Amplification of qPCR reference gene
*yadG*-F	TTATCTGGCGGGATGAAGC	19	qPCR detection of *yadG* transcription level
*yadG*-R	GAATGATGGTGGTGCCTTTG	20	qPCR detection of *yadG* transcription level
*yadH*-F	GCCGCCAGTCATCACCAT	18	qPCR detection of *yadH* transcription level
*yadH*-R	CGCTACCAGCAGCTCTTCAAT	21	qPCR detection of *yadH* transcription level

### 2.2 Major reagents and instruments

Restriction enzymes and T4 DNA ligase were purchased from Takara Bio Inc. The ChIP assay kit was obtained from Sangon Biotech. Reverse transcriptase and SYBR Green qPCR Master Mix were purchased from Wuhan Servicebio.

Antibiotic concentrations were set as follows: ampicillin (Amp, 100μg/mL), kanamycin (Kan, 50?μg/mL), and chloramphenicol (Cm, 50μg/mL). For cultivation of λ*pir* host strains, diaminopimelic acid (DAP) was used at a working concentration of 50μg/mL.

Key instruments included a high-speed refrigerated centrifuge (Eppendorf, Germany), a thermal cycler (Bio-Rad, United States), a real-time PCR system (Applied Biosystems, United States), a gel imaging system (Bio-Rad, United States), and a sonicator (Sonics, United States).

### 2.3 Expression vector construction

The *marA* gene was amplified from *E. coli* K-12 MG1655 genomic DNA using KOD-Plus-Neo high-fidelity DNA polymerase (Toyobo). *Bam*HI and *Hin*dIII restriction sites were introduced at the 5′ ends of the primers for directional cloning. After double digestion with *Bam*HI and *Hin*dIII, the PCR product was ligated into the linearized pET-32a(+) vector. The recombinant plasmid pET-32a-*marA* was transformed into competent *E. coli* BL21(DE3) cells. Positive clones were verified by colony PCR and restriction analysis. The recombinant plasmid was confirmed by Sanger sequencing using specific primers *marA*-F and *marA*-R (Table 2) to cover the full-length *marA* coding region.

### 2.4 Gene deletion

Two strategies were used to construct gene deletion mutants.

(1) Suicide plasmid-mediated homologous recombination was used to generate in-frame deletions of *marA* and *yadG*. Approximately 500-bp upstream and downstream homology arms were amplified and fused by overlap PCR. The fusion construct was designed to remove the complete open reading frame while retaining an uninterrupted reading frame to achieve in-frame deletion.

The fusion fragment was cloned into the suicide vector pDM4 and transferred into donor strain *E. coli* S17-1 *λpir*. Conjugation was performed with the recipient strain *E. coli* K-12. Transconjugants were selected on LB plates containing chloramphenicol (50μg/mL). Positive exconjugants were then screened on LB supplemented with 10% sucrose for *sacB*-based counterselection. Mutants were verified by PCR and DNA sequencing to confirm accurate in-frame gene removal.

(2) λ-Red homologous recombination was applied to construct an in-frame deletion mutant of *yadH*. This system enables targeted gene replacement via recombinase expression. The chloramphenicol resistance cassette flanked by FRT sites was amplified from plasmid pKD3. The PCR product was electroporated into *E. coli* K-12 harboring pKD46. Recombinase expression was induced by 10 mmol/L L-arabinose at 30°C. Mutants were identified and confirmed by PCR and sequencing.

### 2.5 MIC determination

MICs were determined by the broth microdilution method according to CLSI standards. Overnight cultures were adjusted to 0.5 McFarland standard (∼1 × 10^8^ CFU/mL) and diluted 1:200 in cation-adjusted Mueller-Hinton broth (CAMHB; Ca^2+^ 25 mg/L, Mg^2+^ 12.5 mg/L). Final inoculum density was approximately 5 × 10^5^ CFU/mL. Antibiotic concentrations ranged from 0.125 to 512 μg/mL. Plates were incubated statically at 37°C for 18–20 h. The MIC was defined as the lowest drug concentration that inhibited visible growth. Experiments were repeated three times independently. CLSI clinical breakpoints were only cited for descriptive reference in this work rather than for formal resistance/susceptibility categorization, as several tested compounds are not standard clinical antimicrobials designated for *E. coli* susceptibility testing.

### 2.6 Biofilm formation and motility assays

Biofilm formation was quantified using a 96-well plate crystal violet assay. Two microliters of bacterial suspension (0.5 McFarland) was added to wells containing 198 μL LB. Plates were incubated statically at 37°C for 36 h. Floating cells were removed, and wells were washed three times with sterile water. Biofilms were fixed with methanol for 15 min, air-dried, and stained with 1% crystal violet for 8 min. After rinsing, bound dye was solubilized in 200 μL of 33% acetic acid, and absorbance was measured at 590 nm (OD_590_). Biofilm levels were classified according to Stepanović et al. (2000): OD ≤ ODc (non-adherent); ODc < OD ≤ 2 × ODc (weak); 2 × ODc < OD ≤ 4 × ODc (moderate); OD > 4 × ODc (strong). This classification, originally developed for staphylococcal biofilms (Stepanović et al., 2000), has been widely used in *E. coli* biofilm studies (Yamasaki et al., 2015).

Motility was assessed using soft agar plates. The optical density of overnight cultures was adjusted to an OD_600_ of 1.0. Two microliters of culture were spotted onto the center of 0.3% (swimming) or 0.5% (swarming) agar plates. Plates were incubated at 37°C for 24 h, and the diameters of the motility halos were measured. All assays were performed in triplicate.

### 2.7 qRT-PCR assay

Total RNA was extracted from *E. coli* K-12, *E. coli*Δ*marA*, and *E. coli* pET-32a-*marA* using Trizol reagent. Residual genomic DNA was removed using RNase-free DNase I. RNA purity was confirmed using a NanoDrop spectrophotometer, with A260/A280 ratios ranging from 1.8 to 2.0. One microgram of total RNA was reverse transcribed into cDNA using PrimeScript RT Master Mix.

The *gapA* gene served as the internal reference. Relative expression levels of *yadG* and *yadH* were quantified using SYBR Green Master Mix. The cycling conditions were as follows: initial denaturation at 95°C for 5 min; followed by 40 cycles of 95°C for 10 s and 60°C for 30 s. Melting curve analysis was performed to verify amplification specificity.

Three biological replicates were conducted for each sample. Relative gene expression was calculated using the 2^∧^−ΔΔCt method assuming comparable amplification efficiencies between the target and reference genes (Schmittgen and Livak, 2008). Melting curve analysis confirmed single specific products for all primer pairs, with no detectable primer-dimer formation.

### 2.8 EMSA assay

Biotin-labeled probes corresponding to the wild-type (WT) *yadG* promoter and a mutant (Mut) probe with a mutated core sequence were synthesized (Table 2). For each binding reaction, 200 ng of biotin-labeled probe was incubated with 5 μg of purified GST-MarA recombinant protein in a final volume of 20 μL EMSA binding buffer (20 mM HEPES-KOH pH 7.9, 50 mM KCl, 1 mM EDTA, 1 mM DTT, 5% glycerol, and 0.1 μg/μL poly(dI-dC) as non-specific competitor). To confirm binding specificity, competition assays were performed by adding a 10 × molar excess of unlabeled WT probe. Mixtures were incubated at 25°C for 20 min and separated by 5% native polyacrylamide gel electrophoresis (Native PAGE) in 0.5 × TBE buffer at 4°C. DNA was transferred to a nylon membrane via wet blotting and visualized using chemiluminescence to detect protein-DNA complexes.

### 2.9 ChIP-qPCR assays

ChIP assays were performed using the Pierce Magnetic Chromatin Immunoprecipitation Kit. Bacterial cultures (*E. coli* K-12 carrying pGBKT7-*marA*) in mid-log phase were treated with 1% formaldehyde at room temperature for 15 min to crosslink protein-DNA complexes, followed by glycine addition (125 mM, 5 min) to stop the reaction. Cells were collected and resuspended in lysis buffer. Chromatin was digested with Micrococcal Nuclease for 7 min at 37°C to generate fragments of 200–500 bp.

The supernatant was incubated with 2 μg rabbit anti-GST antibody and pre-blocked Protein A/G magnetic beads at 4°C overnight. Beads were washed sequentially with low-salt, high-salt, and LiCl wash buffers. Cross-links were reversed by incubation at 65°C overnight in elution buffer (1% SDS, 0.1 M NaHCO*3*). Purified DNA was recovered and 1% of the input material was carried through the same reversal step for %Input calculation.

Two qPCR targets were analyzed: (i) the *yadGH* promoter region (primers *yadG*-F1/R1, Table 2) and (ii) a non-target control region (*gapA* coding sequence, primers GapA-F/R); the *E. coli* 16S rDNA intergenic region served as an additional negative control. Enrichment was calculated as %Input and normalized to a non-specific mouse IgG control (2 μg per reaction). All ChIP-qPCR experiments were performed with three independent biological replicates.

### 2.10 Statistical analysis

All experiments were performed with at least three independent biological replicates, and each biological replicate contained at least two technical replicates. Data are presented as mean ± standard deviation (SD).

For comparisons involving two groups only (e.g., ChIP-qPCR enrichment relative to IgG control), two-tailed Student’s *t*-test was used.

For comparisons involving three or more groups (e.g., biofilm formation, swimming/swarming motility, and qRT-PCR), one-way analysis of variance (ANOVA) was performed, followed by Tukey’s honest significant difference (HSD) *post-hoc* test for pairwise comparisons. Prior to ANOVA, normality of residuals was assessed using the Shapiro–Wilk test, and homogeneity of variances was verified using Levene’s test. In all cases, the assumptions were met (*P* > 0.05 for both tests).

Antimicrobial susceptibility MIC assays were repeated three independent times to confirm reproducible MIC readouts, and no statistical analyses were applied to MIC data.

Statistical significance was defined as follows: *P < 0.05; **P < 0.01; ***P < 0.001. All statistical analyses were performed using OriginPro 2021 (OriginLab Corporation, Northampton, MA, United States).

## 3 Results

### 3.1 Construction of the pET-32a(+)-*marA* recombinant expression vector

The recombinant expression vector pET-32a(+)-*marA* was successfully constructed. The *marA* gene (approximately 384 bp) was amplified from *E. coli* K-12 MG1655 genomic DNA. The PCR product and the empty pET-32a(+) vector were both digested with *Bam*HI and *Hind*III, ligated, and transformed into *E. coli* BL21 competent cells.

**FIGURE 1:**
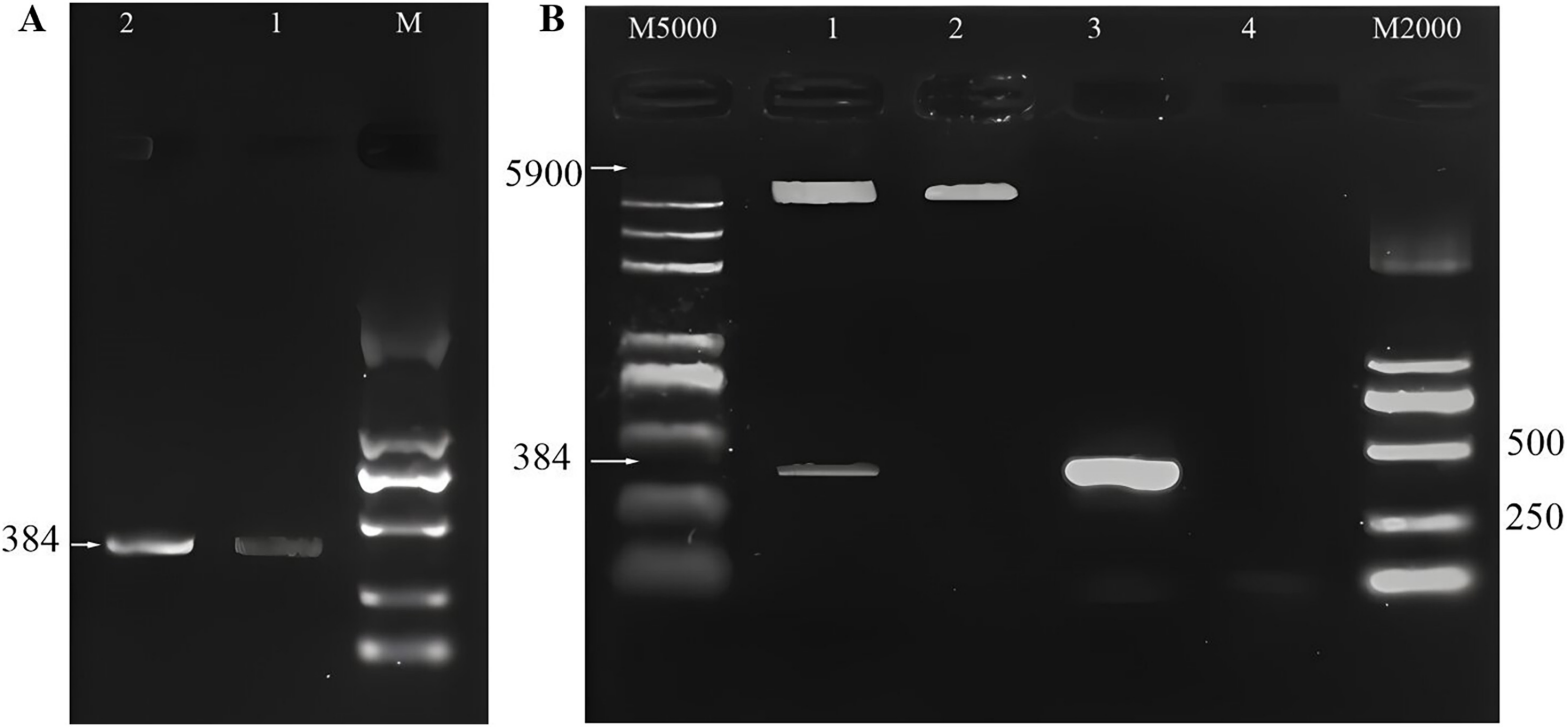
Construction and identification of the recombinant expression plasmid pET-32a(+)-*marA*. M. DNA molecular weight marker. **(A)** PCR amplification of *marA* and restriction digestion of the vector. Lane 1, linearized pET-32a(+) vector after restriction digestion; Lane 2, purified *marA* fragment. **(B)** Identification of recombinant plasmids by colony PCR and restriction digestion. Lanes 1–4, randomly selected colonies. The 5.9 kb band corresponds to the pET-32a(+) vector, and the 384 bp band corresponds to the *marA* gene.

Positive clones were identified by colony PCR and restriction digestion analysis (Figures 1A,B). PCR amplification yielded a specific band of approximately 384 bp, while double digestion released the insert from the vector backbone (approximately 5.9 kb). Single digestion further confirmed the integrity of the vector backbone. Sequencing results showed that the inserted *marA* gene shared 100% identity with the reference sequence in GenBank, with no base mismatches or frameshift mutations detected. These results confirm the successful construction of the pET-32a(+)-*marA* vector for subsequent protein expression and functional studies.

### 3.2 Construction and molecular identification of gene deletion mutants

Deletion mutants of *marA* and *yadG* were constructed in *E. coli* K-12 using suicide plasmid-mediated homologous recombination. A Δ*acrB*Δ*yadG* double deletion mutant was also constructed. The *yadH* deletion was generated using the Red homologous recombination system.

#### 3.2.1 Construction of the *marA* deletion mutant

**FIGURE 2:**
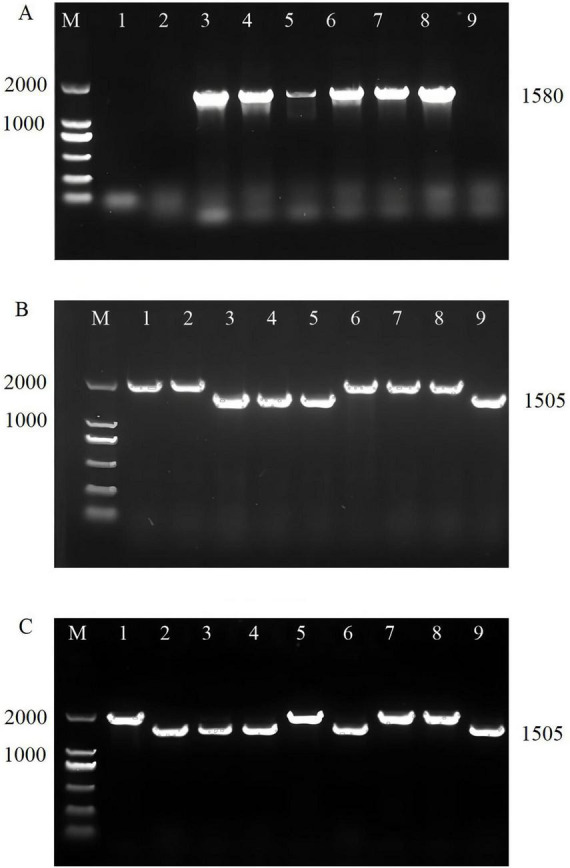
Construction and identification of the Δ*marA* deletion mutant. M. DNA marker. **(A)** Colony PCR identification of recombinant suicide plasmid pDM4-Δ*marA* transformed into donor strain χ7213. Lanes 1–2: negative controls; Lanes 3–8: positive recombinants (1,580 bp). **(B)** Verification of the first-round bacterial conjugation. Lanes 1,2, 6–8: negative controls; Lanes 3–5, 9: positive transconjugants (1,505 bp). **(C)** PCR screening after sucrose counterselection. Lanes 1,5, 7,8: negative controls; Lanes 2–4, 6, 9: confirmed in-frame *marA* deletion mutants (1,505 bp).

The recombinant suicide plasmid pDM4-Δ*marA* was introduced into the DAP-dependent donor strain *E. coli* S17-1 *λpir* via electroporation. Colony PCR results (Figure 2A) showed a specific band of approximately 1,580 bp, confirming successful plasmid construction. Donor cells carrying the recombinant plasmid were conjugated with the recipient strain *E. coli* K-12. After selection on chloramphenicol, positive clones were verified by PCR (Figure 2B), yielding a band of approximately 1,505 bp, indicating successful integration of the plasmid into the chromosome. Exconjugants were then plated on LB medium containing 10% sucrose for counterselection. PCR analysis showed that candidate mutants retained the 1,505 bp band (Figure 2C). Sequencing confirmed the precise deletion of the target region within *marA* without unintended mutations. The *E. coli*Δ*marA* deletion strain was thus successfully constructed.

#### 3.2.2 Construction of the *yadG* deletion mutant

**FIGURE 3:**
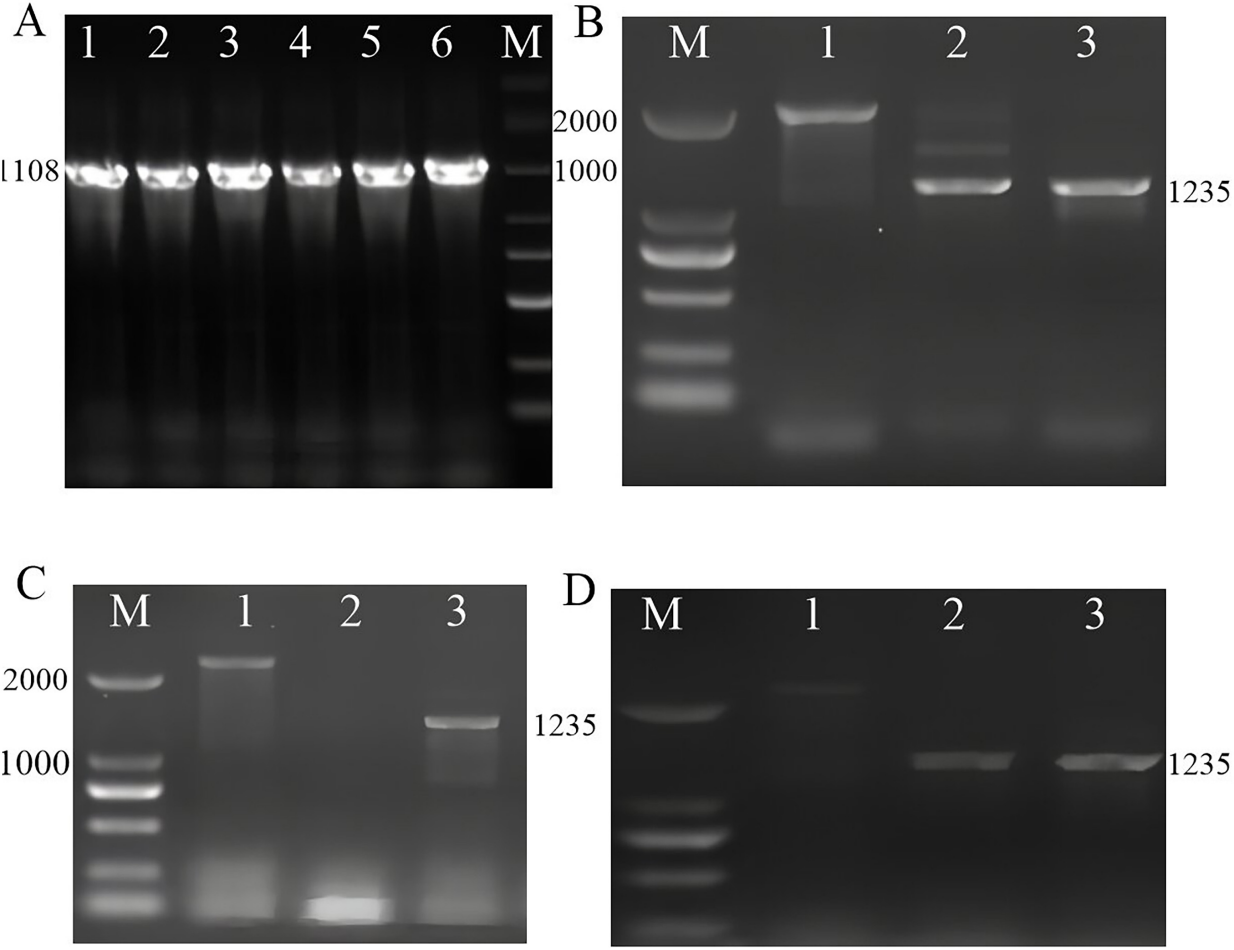
Construction and PCR identification of *E. coli* single Δ*yadG* and double Δ*acrB*Δ*yadG* deletion mutants. M. DNA marker. **(A)** Colony PCR of the recombinant suicide plasmid pDM4-Δ*yadG* in donor strain χ7213. Expected fragment: 1,108 bp. **(B)** PCR verification after the first homologous recombination. Lanes 1: negative controls 2,100 bp; Lanes 2–3: positive transconjugants 1,235 bp. **(C)** PCR identification of the Δ*yadG* single mutant in the *E. coli* K-12 background. Lanes 1: negative controls 2,100 bp; Lanes 3: positive transconjugants 1,235 bp. **(D)** PCR identification of the Δ*acrB*Δ*yadG* double mutant in the *E. coli*Δ*acrB* background. Lanes 1: negative controls 2,100 bp; Lanes 2–3: positive transconjugants 1,235 bp.

The *yadG* deletion mutant was constructed using the same strategy. Colony PCR amplified a specific band of approximately 1,088 bp (Figure 3A), verifying the correct construction of the pDM4-Δ*yadG* suicide plasmid. Following conjugation and first-round recombination, PCR verification (Figure 3B) revealed fragments of 1,100 bp (positive) and 2,100 bp (negative). After sucrose counterselection, PCR identification was performed (Figure 3C). In the *E. coli* K-12 background, the wild-type strain produced a 2,100 bp fragment, while the deletion mutant yielded a shorter 1,500 bp fragment. The deleted fragment size (approximately 600 bp) matched the expectation. The same knockout effect was observed in the Δ*acrB* background (Figure 3D). Sequencing further confirmed the precise deletion of *yadG*. Thus, the Δ*yadG* single mutant and the Δ*acrB*Δ*yadG* double mutant were successfully constructed.

#### 3.2.3 Construction of the *yadH* deletion mutant

**FIGURE 4:**
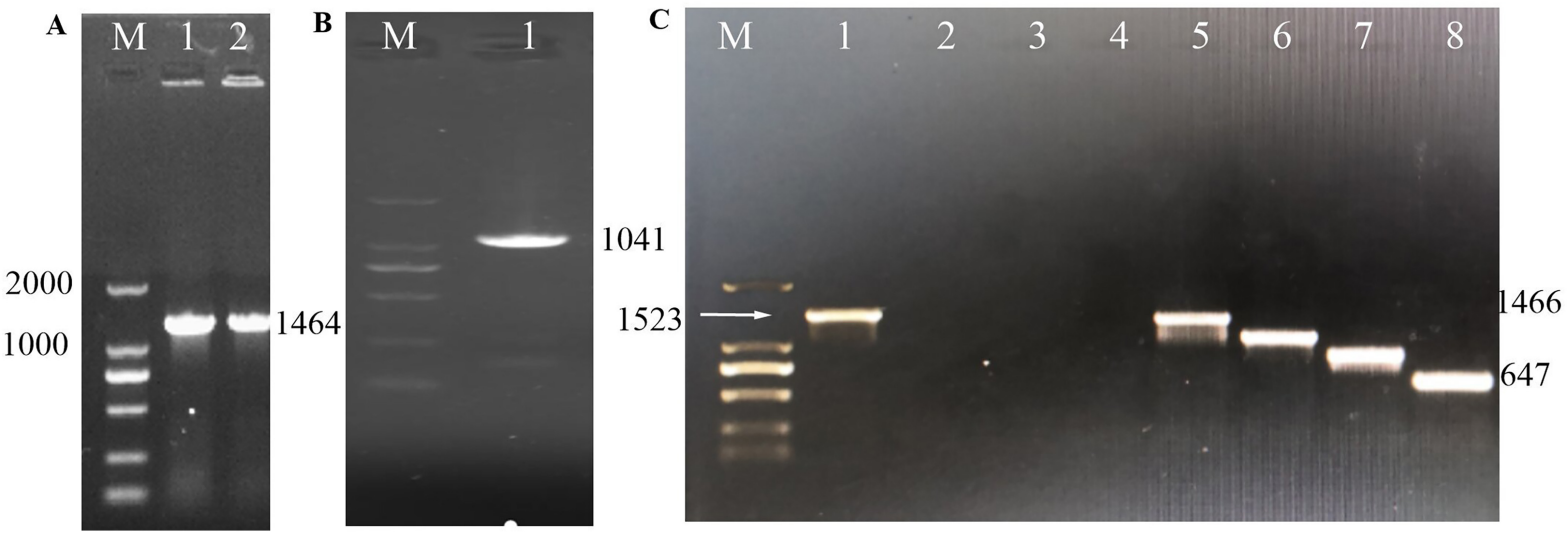
Identification of the targeting fragments and recombinant plasmid during *yadH* gene deletion. M. DNA marker. **(A)** Agarose gel electrophoresis of the pKD46 plasmid, showing the 1,464 kb band in lanes 1–2. The pKD46 plasmid was successfully transformed into the *E. coli* K12 strain. **(B)** PCR amplification of the targeting fragment, yielding a specific 1,041 kb band in lane 2. **(C)** PCR verification of the *yadH* knockout strain. Lanes 1–4: Negative controls of *E. coli* K-12. Amplification was performed using four primer pairs. Only the primers flanking the homologous arms yielded bands, while no bands were observed with the internal gene primers. Lanes 5–8 represent the positive clones of Δ*yadH* verified by amplification with four pairs of primers.

The *yadH* deletion was performed using the Red homologous recombination method. First, the plasmid pKD46, carrying the λ-Red recombinase genes, was transformed into *E. coli* K-12 competent cells (Figure 4A) and incubated at 30°C to induce recombinase expression. The chloramphenicol resistance (*cat*) targeting fragment flanked by FRT sites was amplified from pKD3. The PCR product was digested with *Dpn*I to remove the template plasmid, purified, and electroporated into *E. coli* K-12 carrying pKD46.

Electrophoresis of the targeting fragment revealed a specific band of approximately 1,041 bp (Figure 4B). Positive clones were verified by colony PCR (Figure 4C). Using wild-type *E. coli* K-12 as a negative control, four primer pairs (T-*yadH*-F/R, T-*yadH*-F/Cm-R, Cm-F/T-*yadH*-R, and Cm-F/Cm-R) were used. Positive strains amplified specific fragments (approximately 1,590 bp) containing both *yadH* and the *cat* gene. In contrast, the negative control only produced a band with the T-*yadH*-F/R primers. Sequencing results confirmed the successful knockout of *yadH*.

### 3.3 Effect of the YadGH transporter on biofilm formation and motility

#### 3.3.1 Effect of gene deletions on biofilm formation

**FIGURE 5:**
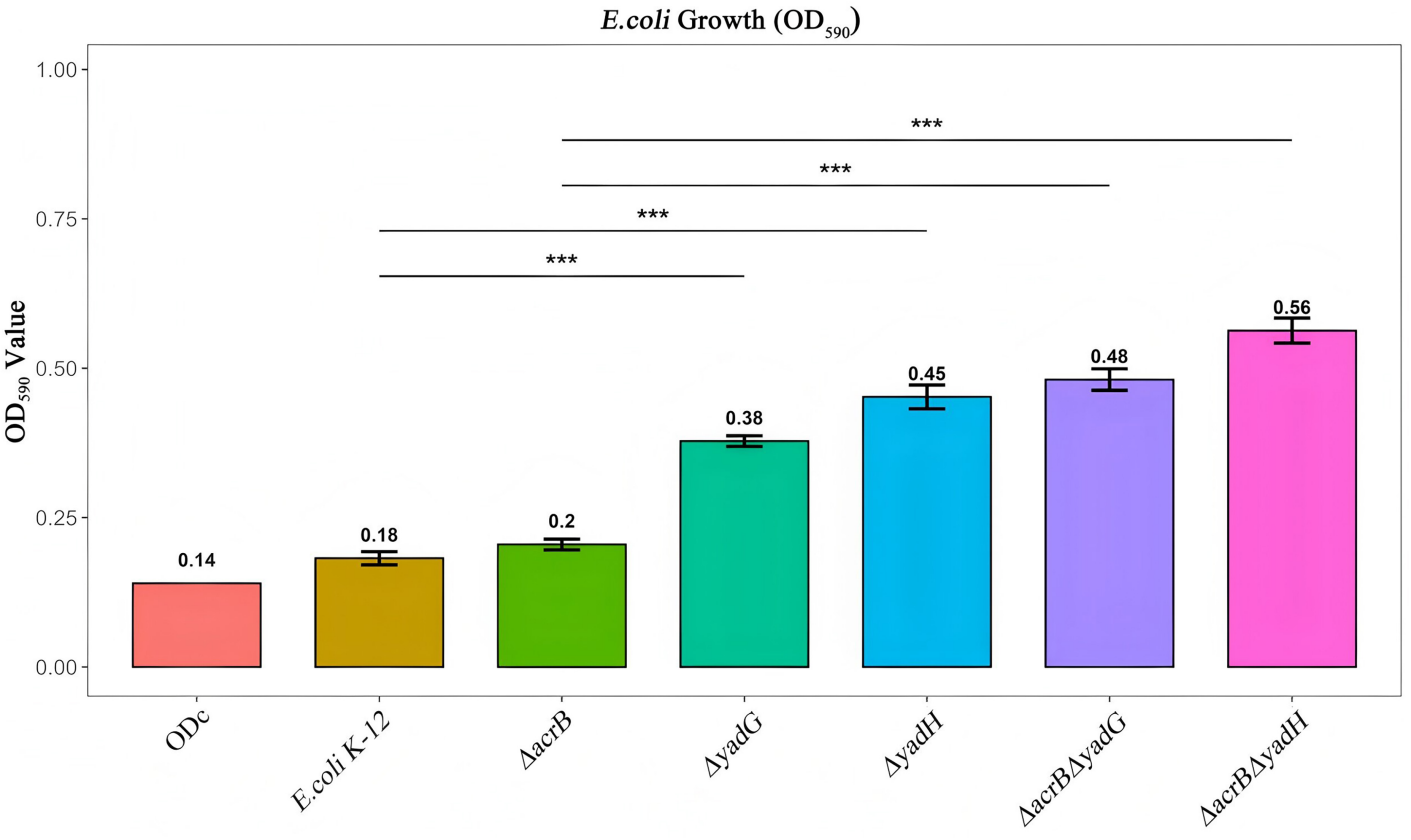
Biofilm formation capacity of wild-type and knockout strains. Bars represent the mean ± SD of three independent experiments. Horizontal axis: Strain types (*E. coli* K-12, Δ*acrB*, Δ*yadG*, Δ*yadH*, Δ*acrB*Δ*yadG*, and Δ*acrB*Δ*yadH*). Vertical axis: Absorbance at 590 nm (OD*590*). Statistical comparisons among strains were performed using one-way ANOVA with Tukey’s HSD *post-hoc* test (*P < 0.05; **P < 0.01; ***P < 0.001). The ODc (cut-off) value was calculated as the mean OD of the negative control plus three times the standard deviation.

Biofilm formation after 36 h of cultivation was measured by crystal violet staining assay (Figure 5). The wild-type *E. coli* K-12 yielded an OD*590* of 0.182 ± 0.011. Deletion of *acrB* raised this value to 0.205 ± 0.009 (*P* < 0.05). More substantial increases were observed upon deletion of *yadG* or *yadH* (*P* < 0.01), with Δ*yadH* (0.452 ± 0.020) producing more biofilm than Δ*yadG* (0.387 ± 0.015). Combined deletions further enhanced biofilm formation: the Δ*acrB*Δ*yadG* double mutant reached 0.481 ± 0.018, and the Δ*acrB*Δ*yadH* double mutant reached 0.563 ± 0.022, both significantly higher than their respective single mutants (*P* < 0.01). Based on standard classification criteria, the wild-type and Δ*acrB* strains were weak biofilm producers (Yamasaki et al., 2015), whereas the Δ*yadG* and Δ*yadH* single mutants were moderate producers. The double mutants exhibited greatly improved biofilm-forming ability. All pairwise comparisons were performed using one-way ANOVA with Tukey’s HSD *post-hoc* test (see section 2.10).

#### 3.3.2 Motility analysis

##### 3.3.2.1 Swimming motility

**FIGURE 6:**
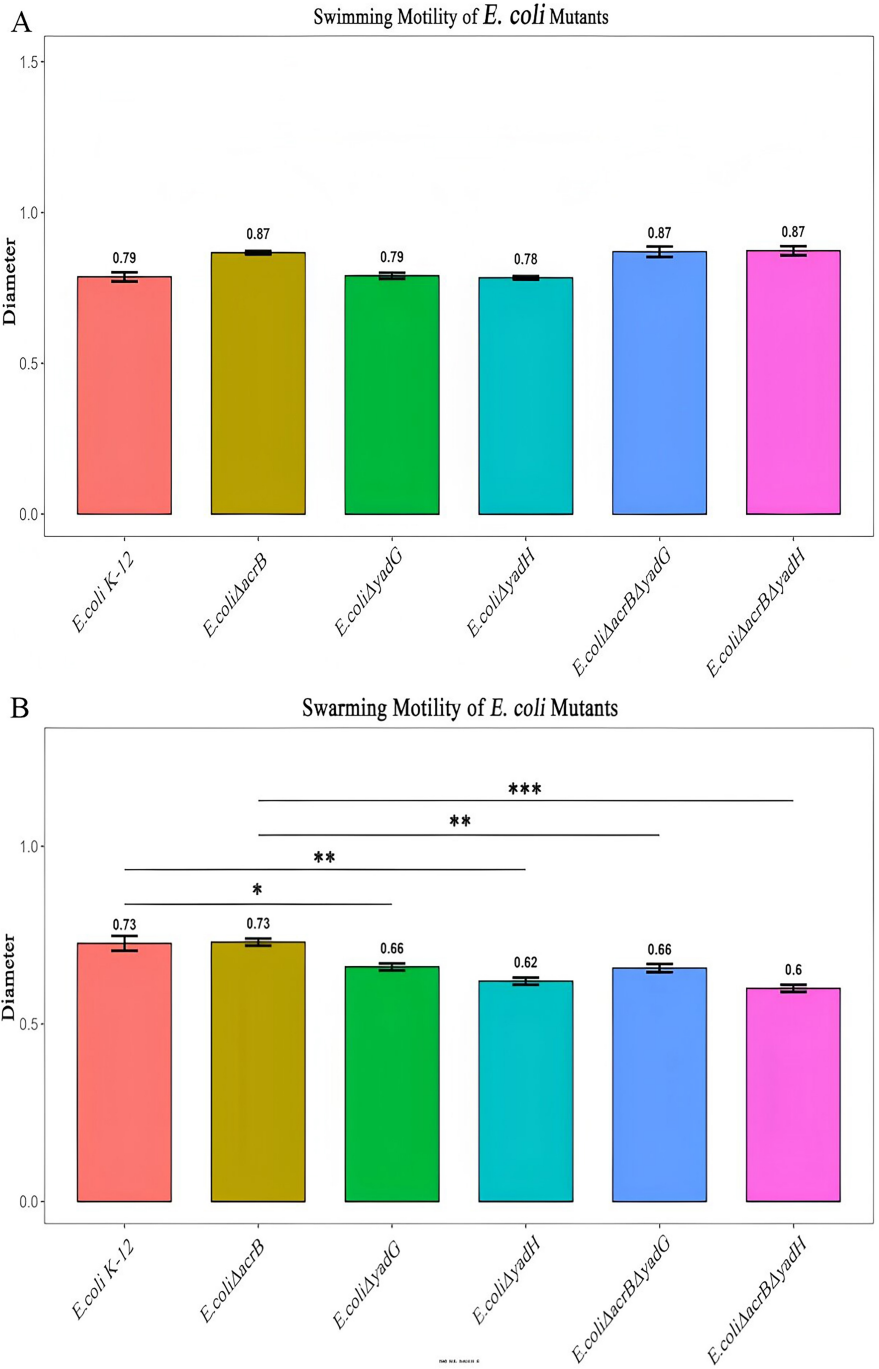
Motility assays of different gene deletion mutants. **(A)** Swimming motility assay. Two microliters of bacterial culture (OD*600* = 1.0) were spotted onto 0.3% soft agar plates. Plates were incubated at 37°C for 24 h. The diameter of the motility halo was measured in centimeters (cm). **(B)** Swarming motility assay. Two microliters of bacterial culture (OD*600* = 1.0) were spotted onto 0.5% soft agar plates. Plates were incubated at 37°C for 24 h. The diameter of the swarm expansion zone was measured in centimeters (cm). Data are mean ± SD from three independent biological replicates. Statistical comparisons among strains were performed using one-way ANOVA with Tukey’s HSD *post-hoc* test (*P < 0.05; **P < 0.01; ***P < 0.001).

In low-viscosity medium, the Δ*acrB* strain exhibited a larger swimming diameter (0.85 ± 0.02 cm) compared to the wild-type (0.78 ± 0.03 cm, *P* < 0.05), indicating that AcrB inhibits flagellar movement. The Δ*yadG* and Δ*yadH* strains showed no significant difference from the wild-type. Double deletion strains also did not differ from the Δ*acrB* strain (Figure 6A). These results indicate that the YadGH transporter does not regulate swimming motility.

##### 3.3.2.2 Swarming motility

In high-viscosity medium, deletion of YadGH reduced swarming. The Δ*yadG* strain showed a reduced swarming diameter (0.66 ± 0.03 cm) compared to the wild-type (0.72 ± 0.03 cm, *P* < 0.05). The Δ*yadH* strain showed an even greater reduction (0.64 ± 0.02 cm, *P* < 0.01), suggesting that YadH is a positive regulator of swarming. The Δ*acrB*Δ*yadH* double deletion strain exhibited the smallest swarming diameter (0.61 ± 0.02 cm), representing an additive inhibitory effect compared to the single deletion strains (*P* < 0.001, Figure 6B). Visual inspection of the plates showed that the wild-type spread evenly with regular edges. The Δ*yadH* strain showed limited spread with rough, sparse edges. The Δ*acrB*Δ*yadH* double deletion strain barely spread. Collectively, these findings suggest that YadGH and AcrB cooperate to promote swarming motility.

Statistical significance for all motility comparisons was assessed by one-way ANOVA with Tukey’s HSD *post-hoc* test.

### 3.4 Effect of the YadGH transporter on MICs

The broth microdilution method was used to determine the susceptibility of wild-type *E. coli* K-12 and its derivatives to 15 clinical antibiotics. Strains included single deletions of *yadG* or *yadH*, the Δ*acrB* strain, and the corresponding double deletion mutants. All experiments were performed in triplicate. Compared to the wild-type, single deletions of *yadG* or *yadH* caused minimal changes in MIC values (less than one dilution step). This suggests that the YadGH transporter contributes little to broad-spectrum antibiotic resistance. The only statistically significant change occurred with the macrolide antibiotic tylosin (Table 3). In the wild-type background, deletion of *yadG* or *yadH* did not alter the tylosin MIC (512 μg/mL). However, in the Δ*acrB* background, further deletion of *yadG* or *yadH* significantly increased sensitivity. In the Δ*acrB* background, further deletion of *yadG* reduced the tylosin MIC from 256 to 64 μg/mL (four-fold) (*P* < 0.01). The *yadG* and *yadH* deletion strains showed identical phenotypes, indicating they form a functional unit where loss of either subunit inactivates the system. The YadGH transporter appears to contributes to the export of macrolides like tylosin, particularly when AcrB is absent.

**TABLE 3:** Antimicrobial susceptibility profiles of wild-type and mutant *E. coli* strains.

Antibiotic	MIC (μ g/mL)							
	*E. coli* K-12	pET-32a-*marA*	*E. coli* Δ *marA*	*E. coli* Δ *yadG*	*E. coli* Δ *yadH*	*E. coli* Δ *acrB*	*E. coli*Δ *acrB* Δ *yadG*	*E. coli*Δ *acrB* Δ *yadH*
Erythromycin	16	16	1	16	16	8	1	1
Tylosin	512	512	64	512	512	256	64	64
Azithromycin	1	1	0.25	1	1	0.5	0.25	0.25
Levofloxacin	0.0312	0.0312	0.0078	0.0312	0.0312	0.0078	0.0039	0.0039
Daunorubicin	64	64	0.5	64	64	0.5	0.5	0.5
Doxorubicin	64	64	0.5	64	64	0.5	0.5	0.5
Acriflavine	16	16	16	16	16	16	16	16
Cefoperazone	0.5	0.5	0.5	0.5	0.5	0.5	0.5	0.5
Tobramycin	4	4	4	4	4	2	2	2
Polymyxin B	2	2	2	2	2	1	1	1
Tetracycline	0.5	0.5	0.5	0.5	0.5	0.125	0.125	0.125
Rifampicin	8	8	8	8	8	4	4	4
Chloramphenicol	>1,024	>1,024	1,024	>1,024	>1,024	1,024	1,024	1,024
Amphotericin B	>1,024	>1,024	1,024	>1,024	>1,024	>1,024	>1,024	>1,024
Spectinomycin	16	16	16	16	16	16	16	16

Deletion of *marA* alone yielded erythromycin and tylosin MIC values of 1 and 64 μg/mL, respectively, matching results from the Δ*acrB*Δ*yadG* and Δ*acrB*Δ*yadH* double mutants (Table 3). These matching resistance phenotypes confirm that YadGH acts as the key MarA-dependent mediator of macrolide resistance when AcrB is absent.

### 3.5 qRT-PCR analysis

Total RNA was extracted using Trizol reagent and reverse transcribed into cDNA. The *gapA* gene was used as the internal reference. Relative expression levels of *yadG* and *yadH* were measured using SYBR Green detection. Three biological replicates were performed for each strain.

**FIGURE 7:**
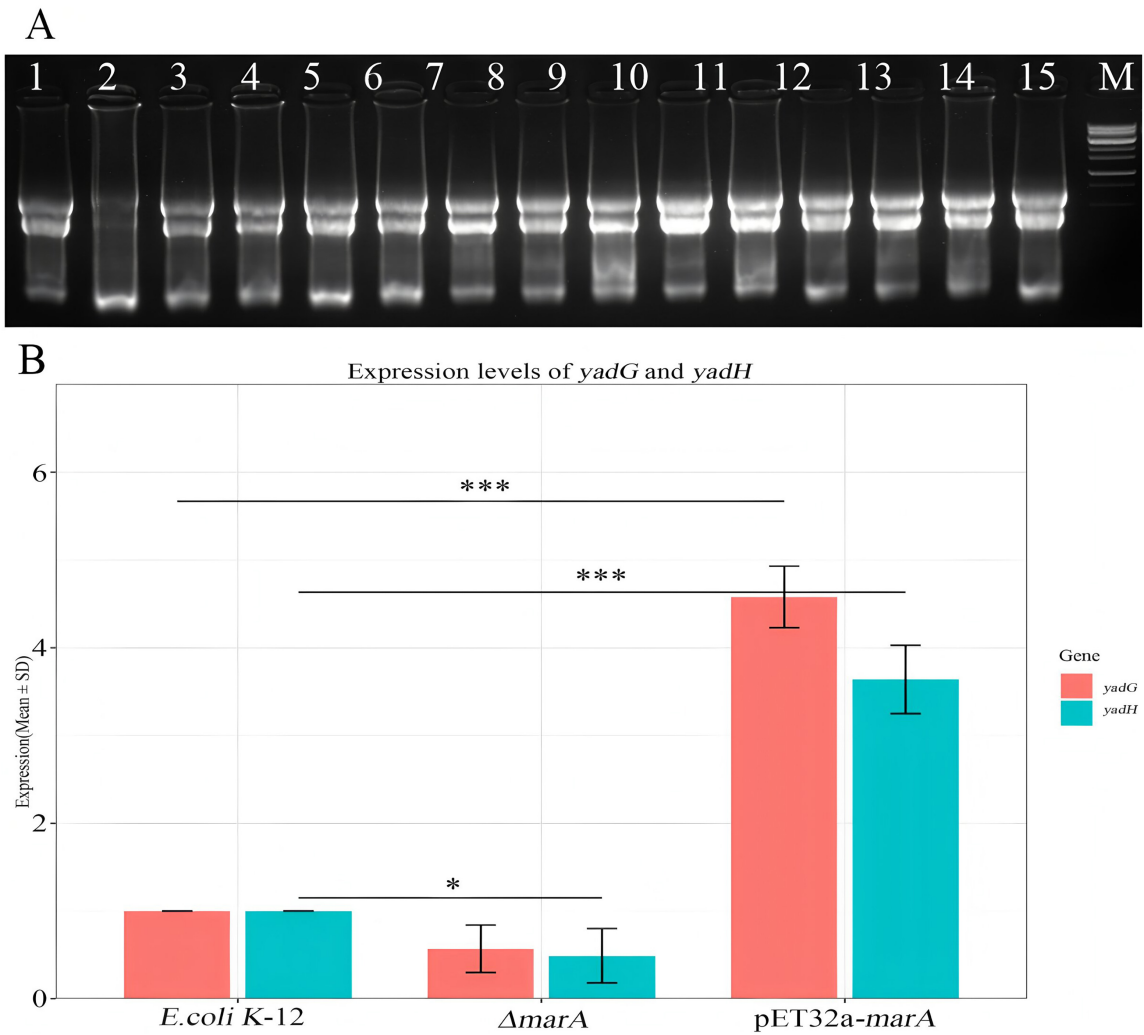
Effects of marA overexpression on the expression of *yadG* and *yadH* in *E. coli*. **(A)** Total RNA electrophoresis profiles for samples 1-15. All samples displayed uniform smears without obvious genomic DNA contamination, confirming qualified RNA quality. **(B)** Relative quantification of *yadG* and *yadH* mRNA levels by quantitative real-time PCR (qRT-PCR). Data are mean SD from three independent experiments. Statistical analysis was performed using one-way ANOVA with Tukey’s HSD *post-hoc* test (*P<0.05; **P<0.01; ***P<0.001) compared to the *E. coli* K-12 control.

RNA integrity and genomic DNA contamination were assessed by 1.5% agarose gel electrophoresis (Figures 7A,B). One sample (sample 2) showed slight degradation and was discarded; fresh RNA was re-isolated. The remaining 14 samples displayed clear 23S, 16S, and 5S rRNA bands, without visible smearing or genomic DNA contamination, confirming that RNA quality met qPCR requirements.

qPCR results (Figure 7B) showed that relative to wild-type *E. coli* K-12, expression of *yadG* and *yadH* was significantly downregulated in the Δ*marA* deletion strain (*P* < 0.05). In the pET-32a-*marA* overexpression strain, both genes were significantly upregulated (*P* < 0.01). No significant change was observed in the Δ*acrB* strain. These results suggest that MarA positively regulates transcription of *yadG* and *yadH*.

Statistical comparisons among the four strains (K-12, Δ*marA*, Δ*acrB*, and pET-32a-*marA*) were performed using one-way ANOVA with Tukey’s HSD *post-hoc* test; the wild-type K-12 strain served as the control group for pairwise comparisons.

### 3.6 EMSA assay

Biotin-labeled probes for the *yadGH* promoter and a mutant probe were synthesized. Purified MarA protein was incubated with the probes in EMSA binding buffer. Protein-DNA complexes were separated by native PAGE, transferred to membranes, and detected by chemiluminescence.

#### 3.6.1 Verification of GST-MarA protein expression

**FIGURE 8:**
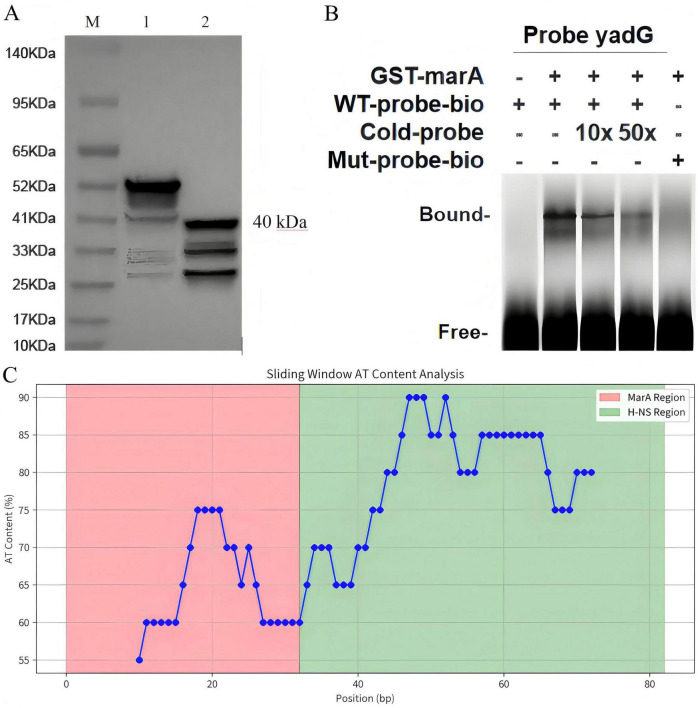
*In vitro* specific binding of GST-MarA protein to the *yadG* promoter. **(A)** Western blot analysis using an anti-GST antibody. Lane 1: protein marker; Lane 2: irrelevant protein; Lane 3: purified GST-MarA recombinant protein (∼40 kDa). **(B)** Electrophoretic mobility shift assay (EMSA). Lane 1: Biotin-labeled wild-type probe (WT-bio); Lane 2: WT-bio + GST-MarA, showing the shifted complex (Bound); Lanes 3–4: Competition assays with 10 × and 50 × excess unlabeled probe, resulting in disappearance of the bound complex; Lane 5: Mutant probe (Mut-bio), showing no binding. GST-MarA specifically binds to the *yadG* promoter. **(C)** Sliding window AT content analysis of the *yadGH* promoter region.

After prokaryotic induction, affinity purification, and Western blotting, the GST-MarA recombinant protein appeared as a single clear band at the expected molecular weight, with no visible contamination. This confirmed correct expression and sufficient purity for EMSA experiments (Figure 8A).

#### 3.6.2 EMSA confirmation of specific binding between MarA and the *yadG* promoter

EMSA revealed a distinct mobility shift for the biotin-labeled wild-type *yadG* promoter probe (P) after incubation with GST-MarA (lanes 1–2, Figure 8B). Addition of 10- or 50-fold molar excess of unlabeled competitor DNA diminished the shifted bands in a concentration-dependent manner (lanes 3–4), confirming a specific and reversible DNA-protein interaction. EMSA mapped the MarA-binding site to a region spanning positions −1,579 to −1,546 bp upstream of the *yadG* start codon. To validate binding specificity, a mutant probe (MP) was generated. The core MarA-binding motif was altered. Compared to the wild-type sequence (WT: 5′-…GCCATAAAGAG…-3′’), the mutant sequence (Mut: 5′-…CATGGACATAAAGAG…-3′) substitutes four nucleotides within the core motif. This mutant probe failed to produce a mobility shift (lane 5). This result shows that MarA binding to the *yadG* promoter strictly requires this core sequence.

Further sequence analysis of the MarA-binding region (Figure 8C) revealed that the 20-bp sequence flanking the core motif (5′-TGTATTGTTAA-3′) has an intermediate AT content of 56.25%, whereas an adjacent downstream 50-bp stretch features a markedly elevated AT content of 80.00%. The functional relevance of this AT-rich region is currently unknown and is further considered in the Discussion.

To characterize the promoter architecture of the *yadGH* operon, we scanned the 500 bp region immediately upstream of the *yadG* start codon for σ^7^0-dependent promoter elements. No canonical −35/−10 consensus sequences were identified within this region. All coordinates for the MarA binding site are reported relative to the *yadG* translation initiation codon (ATG). As annotated in RegulonDB (v11.0), the *yadGH* operon currently has no experimentally validated promoter or transcription start site (TSS).

### 3.7 ChIP-qPCR assay

**FIGURE 9:**
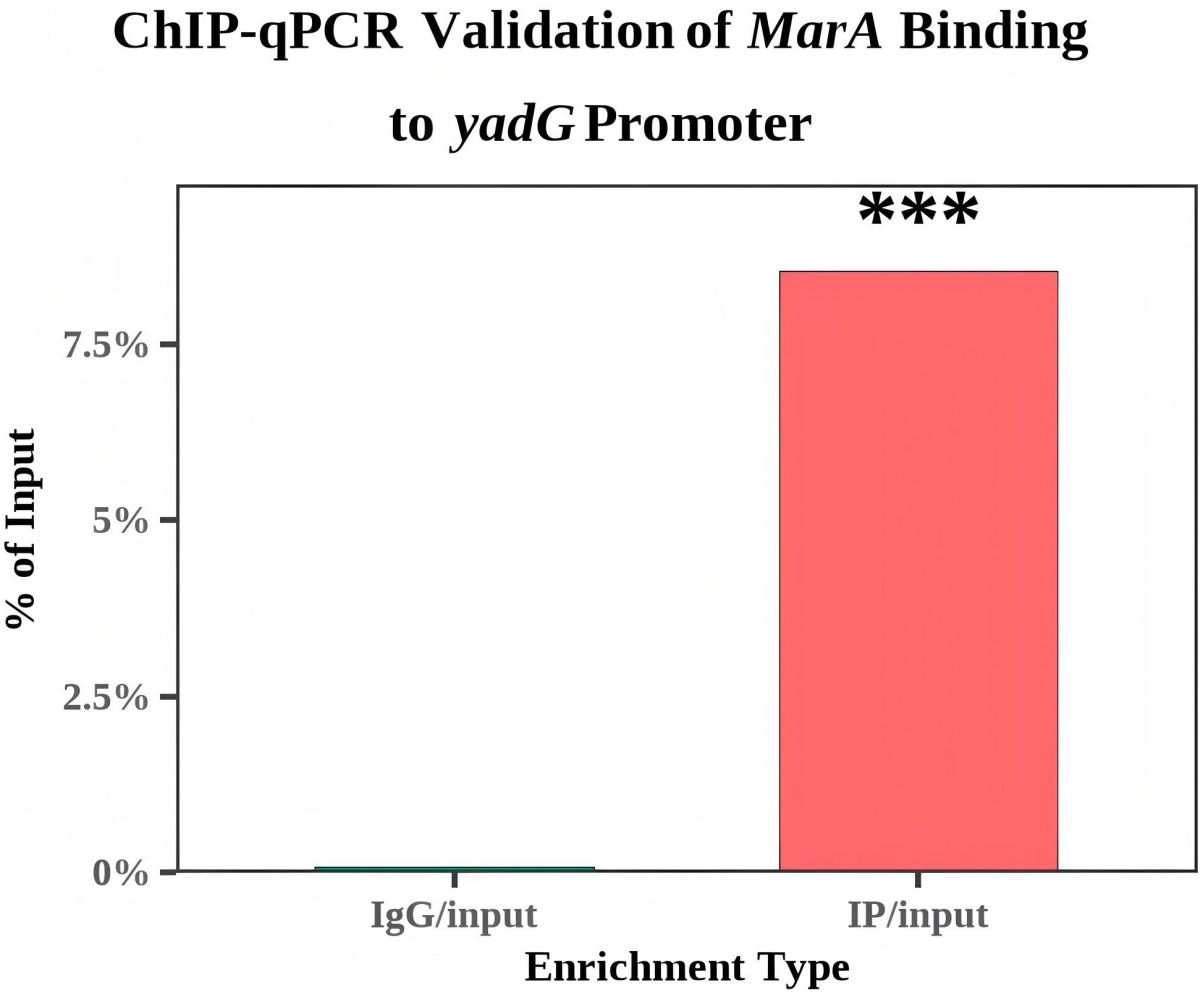
MarA binds to the *yadGH* promoter *in vivo*. ChIP-qPCR analysis of the *yadGH* promoter region. Data are presented as the percentage of input, shown as mean ± SD from three independent biological replicates. The *yadGH* promoter fragment was significantly enriched in the GST-MarA immunoprecipitated sample compared to the negative control (IgG). Statistical analysis was performed using two-tailed Student’s *t*-test (*P < 0.05; **P < 0.01; ***P < 0.001).

ChIP-qPCR was used to test whether MarA binds to the *yadGH* promoter *in vivo*. Chromatin was immunoprecipitated using an anti-GST antibody. qPCR results showed significant enrichment of the *yadGH* promoter region in the precipitated DNA (*P* < 0.001, Student’s *t*-test, relative to the IgG control; Figure 9). No obvious enrichment was detected in the input DNA or IgG control groups. Thus, MarA binds to the *yadGH* promoter *in vivo* and positively regulates its transcription.

## 4 Discussion

This study integrates transcriptional regulation analysis, *in vitro* and *in vivo* binding validation, and phenotypic characterization to identify the ABC efflux transporter YadGH as a novel direct target of the global stress regulator MarA. We mapped the MarA binding site within the *yadGH* promoter using EMSA with mutant probes. Notably, DNA pull-down screening suggested that the YadGH promoter region may also be recognized by the global silencer H-NS, raising the possibility of a competitive regulatory model similar to the *marRAB* operon (Wang et al., 2024). Although deletion of YadG or YadH alone had little effect on conventional antibiotic susceptibility, its cooperative efflux of tylosin and erythromycin in the absence of the major efflux pump AcrB, together with its effects on biofilm formation and swarming motility, suggests that YadGH functions in bacterial physiology beyond conventional drug resistance. Below, we discuss the transcriptional regulation of the MarA-YadGH axis, the functional division of efflux, and the multidimensional roles of YadGH in bacterial adaptation.

### 4.1 MarA binds to the *yadGH* promoter and positively regulates its transcription

We identified the ABC efflux transporter YadGH as a new transcriptional target of the global stress regulator MarA in *E. coli* K-12. ChIP-qPCR results showed that the *yadGH* promoter region was significantly enriched in GST-MarA immunoprecipitates (Figure 9). EMSA demonstrated specific binding between the two *in vitro*. Using mutant probes, we mapped the core MarA binding motif (5′-TGTATTGTTAA-3′) to a region spanning positions -1.5 kb upstream of the *yadG* start codon; mutation of this core sequence completely abolished binding. All coordinates for the MarA binding site are reported relative to the *yadG* translation initiation codon (ATG), as no experimentally validated transcriptional start site (TSS) is currently annotated for this operon in RegulonDB (v11.0; promoters: 0). To assess the promoter context of this distal binding site, we scanned the 500 bp region immediately upstream of the *yadG* ATG for σ^70^-dependent promoter elements; no canonical −35/−10 consensus sequences were identified within this region. The identification of MarA binding to a degenerate marbox upstream of the *yadGH* operon and MarA-dependent induction of *yadGH* transcription extends the MarA regulon beyond previously characterized targets such as *mlaFEDCB* and *xseA* (Sharma et al., 2017), representing a context-dependent addition to the MarA-controlled network in *E. coli*.

This motif does not fully match the canonical marbox (5′-WWMARAACAYYTMMWW-3′), indicating it is a degenerate variant. Nevertheless, EMSA and qRT-PCR results confirmed that MarA binding correlates with increased *yadGH* transcription. Studies by Martin et al. (1999) showed that MarA can recognize degenerate, truncated marbox variants, and its binding relies on shape complementarity rather than strict sequence conservation, which is consistent with our observations (Martin et al., 1999).

Notably, the *yadGH* promoter region is composed of two distinct regions: the MarA core binding region itself has a lower AT content (56.25%), whereas the immediate downstream 50-bp sequence has a high AT content of 80.00% (Figure 8C). The MarA binding site is located approximately 1.5 kb upstream of the *yadG* ATG, an atypical distant position that requires further analysis. Most characterized MarA-regulated loci, including *marRAB* and *micF*, contain binding motifs within 100–200 bp of the TSS. Previous studies have demonstrated that MarA can drive transcription from far-upstream regions via DNA looping, a mechanism prevalent at promoters under H-NS-mediated repression (Martin et al., 1999; Sharma et al., 2017). This established model provides a reasonable explanation for the weak, non-canonical promoter we identified upstream of *yadG*, as such promoters generally require an activator to sustain robust transcription. The AT-rich sequence downstream of the MarA motif further suggests a potential role for H-NS in this regulatory region: classic studies have shown that H-NS nucleates at high-affinity, AT-rich sites and spreads laterally to form silencing complexes (Bouffartigues et al., 2007; Lang et al., 2007). We emphasize that this model remains preliminary, given the absence of direct *in vivo* evidence for H-NS occupancy at this locus.

Based on these observations, the co-enrichment of H-NS at the *yadGH* promoter observed in our DNA pull-down assays, alongside the AT-rich sequence located downstream of the MarA binding motif, leads us to propose a testable hypothesis: H-NS likely maintains basal transcriptional silencing of *yadGH*, and MarA relieves this repression to drive transcription. Drawing on well-documented H-NS-dependent regulatory loci (Bouffartigues et al., 2007; Lang et al., 2007), we put forward a tentative mechanistic model. In this scenario, H-NS nucleates on the downstream AT-rich stretch and spreads along DNA to block the MarA binding site, whereas MarA binding may sterically hinder further assembly of H-NS filaments. Importantly, we stress that this model is purely speculative. Our data only verify MarA promoter binding and MarA-dependent induction of *yadGH*; we lack *in vivo* proof of H-NS occupancy at this locus, H-NS-mediated repression of *yadGH*, or direct competitive binding between MarA and H-NS. Future work including H-NS ChIP-qPCR, transcriptional assays in an *hns* deletion strain, and mutagenesis of the AT-rich region with reporter constructs will be required to directly validate this hypothesis.

### 4.2 YadGH as an auxiliary efflux pump in the absence of AcrAB-TolC

Based on the competitive antagonism regulatory hypothesis of H-NS and MarA proposed in section 4.1, *yadGH* expression is tightly restricted, suggesting that it may serve as an auxiliary system to avoid unnecessary energy expenditure under normal conditions. In agreement, deletion of *yadG* or *yadH* alone did not significantly change MICs for most conventional antibiotics (β-lactams, fluoroquinolones, aminoglycosides). This is consistent with functional redundancy among efflux systems in Gram-negative bacteria: the high-capacity major efflux channel AcrAB-TolC handles most drug efflux, masking the contribution of auxiliary system (Nikaido and Takatsuka, 2009; Cudkowicz and Schuldiner, 2019).

However, when the major channel is impaired (Δ*acrB* background), the physiological role of YadGH becomes apparent. In the Δ*acrB* background, deletion of *yadGH* further sensitized cells to macrolides, particularly tylosin and erythromycin (MIC reduced from 256 to 64 μg/mL, a four-fold difference; Table 3). This consistent shift across independent replicates indicates that YadGH contributes a residual? efflux capacity. Macrolide antibiotics generally have limited outer membrane permeability in Gram-negative bacteria (Klobucar et al., 2022; Zhou et al., 2025), so even a small increase in intracellular drug accumulation can be lethal. Given the elevated intracellular drug accumulation in the Δ*acrB* background, the modest efflux activity of YadGH may be sufficient to lower intracellular drug concentrations and produce a significant phenotypic rescue. This suggests that YadGH is not a major efflux pump but acts as an auxiliary system when the primary channel is compromised, consistent with the hierarchical organization of efflux systems in Gram-negative bacteria in which minor pumps provide residual protection upon loss of the major pump (Nikaido, 2009; Blair et al., 2015).

YadGH does not appear to function as a broad-spectrum resistance pump. In line with this idea, prior work demonstrated the *yadG* promoter is strongly and specifically induced by nitroaromatic compounds (TNT, 1,3-DNB; EC_50_ ≈ 6.5 mg/L), and this element has been utilized to construct TNT biosensors (Tan et al., 2015). Combining our data showing MarA-driven *yadGH* transcription with its minor impact on macrolide susceptibility (tylosin, erythromycin), the collective evidence from published work and our current study indicates the primary physiological function of the MarA-YadGH axis is protection against environmental toxicants rather than clinical antibiotics.

This physiological interpretation is further supported by additional functional properties of YadG. As an efflux pump component, it mediates transmembrane transport of intracellular small molecules and contributes to carbon metabolism homeostasis in *E. coli* (Moussatova et al., 2008; Saier et al., 2016). Specifically, YadG has been shown to interact with the glycolytic enzyme enolase and glutamate decarboxylase B (GadB) in a protein-enzyme interaction (PEI) network, suggesting a regulatory role in both glycolysis and L-glutamate degradation pathways (Chowdhury et al., 2021; Chowdhury et al., 2025). Deletion of this gene increases susceptibility to X-ray and UV irradiation, and impairs the cellular response to radiation-induced DNA damage (Sargentini et al., 2016). These diverse physiological properties reinforce the view that the MarA-YadGH axis has evolved primarily to cope with various environmental stresses, rather than serving as a dedicated clinical antibiotic resistance mechanism.

Based on these results, we tentatively propose that exposure to nitroaromatic pollutants in soil or water induces MarA, thereby driving *yadGH* expression to alleviate specific chemical stresses. Although tylosin, a veterinary macrolide, represents an environmentally relevant selective pressure, the narrow substrate spectrum of YadGH is consistent with its regulatory logic as an auxiliary system. Thus, YadGH functions as a specialized defense tool that is selectively activated under particular environmental stresses, rather than a routine, broad-spectrum efflux weapon.

### 4.3 Phenotypic observations of YadGH deletion on biofilm and swarming motility and potential links to cellular metabolism

The *yadH* deletion strain forms markedly thicker biofilms (Figure 5) and displays attenuated swarming motility relative to the wild type (Figure 6). These phenotypic differences demonstrate that loss of YadGH correlates with altered biofilm assembly and swarming capacity. It cannot yet be resolved whether these shifts arise from direct YadGH-mediated lifestyle control, or as secondary physiological disturbances triggered by the absence of this efflux transporter. In *E. coli*, the balance between motility and biofilm development is governed by two master regulators: the flagellar transcriptional activator FlhDC and curli biogenesis factor CsgD (Sharma and Bearson, 2013). H-NS further suppresses *csgD* transcription via chromatin-level silencing (Dorman, 2024). At present, we have no experimental evidence linking YadGH to either of these canonical regulatory cascades.

To generate plausible mechanistic clues for the observed phenotypes, we referenced published high-throughput proteomic interactome data. These large-scale screens detect physical binding between YadG and two metabolic proteins: glycolytic enolase (Eno), and glutamate decarboxylase GadB, a key enzyme required for glutamate-dependent acid stress tolerance (Chowdhury et al., 2021; Chowdhury et al., 2025). Enolase has been reported to exert secondary moonlighting functions that modulate biofilm formation across multiple bacterial species (Kumar et al., 2023), while GadB serves as a central effector of acid resistance pathways (Ma et al., 2003).

Importantly, all documented YadG-Eno and YadG-GadB interactions originate from untargeted proteomic surveys and have not been validated specifically in the context of YadGH efflux function. Any model positing that YadGH tunes Eno enzymatic activity or modulates GadB-dependent acid survival remains purely hypothetical.

Further dedicated experimentation is required to move beyond correlative phenotypic trends and dissect the causal mechanisms driving biofilm and swarming defects in the Δ*yadH* strain. We outline three complementary experimental avenues for future validation: (i) Quantify transcript levels of flagellar genes (*flhDC*, *fliC*) and curli regulatory factors (*csgD*, *csgA*) in the Δ*yadH* background, to test whether impaired swarming coincides with disrupted expression of well-established motility and biofilm pathways; (ii) Measure intracellular pH homeostasis and acid stress survival rates, to probe whether loss of YadGH interferes with GadB-mediated acid resistance; (iii) Perform *in vivo* co-immunoprecipitation and fluorescence localization microscopy to verify physical or functional interactions between YadGH and Eno inside bacterial cells. Such assays will enable us to distinguish direct regulatory effects of YadGH from indirect metabolic perturbations that secondarily shape biofilm and swarming phenotypes.

### 4.4 Evolution of operon annotation and confirmation of independent transcription units

The results above show that YadGH may influence central carbon metabolism and lifestyle switching via H-NS antagonism and YadG-Eno interactions. This raises a question: does YadGH have independent transcriptional control? Earlier studies suggested *yadG* and *yadH* were part of a large operon with the *yadC-yadN* chaperone-usher fimbrial genes (the Yad fimbrial operon). This implied co-transcription and unified regulation of the YadGH efflux system and Yad fimbriae (Larsonneur et al., 2016).

However, RegulonDB (v11.0) and EcoCyc (v27.0) now classify *yadG-yadH* as a bicistronic operon with its own promoter, separate from *yadC-yadN*. Consistent with this, ChIP-qPCR and EMSA demonstrate MarA binding to a motif within the *yadGH* promoter and activation of transcription. Additionally, the *yadGH* promoter was enriched for H-NS in DNA pull-down assays, consistent with an H-NS-silenced/MarA-derepressed unit (Dorman, 2004). Whether MarA binding drives co-transcription of *yadG* and *yadH* remains to be demonstrated experimentally, e.g., by RT-PCR across the intergenic region.

Although our qRT-PCR data demonstrate that *yadG* and *yadH* are co-regulated by MarA (both genes showed consistent up- and down-regulation in response to MarA levels), these data do not directly prove co-transcription. Such evidence would require RT-PCR across the *yadG-yadH* intergenic region to detect a single transcript spanning both genes. Therefore, we rely on the RegulonDB/EcoCyc annotations for the operon structure, while noting that the co-regulated expression pattern is consistent with, but not conclusive for, the bicistronic operon model.

This independence from the fimbrial genes has important physiological implications. It allows bacteria to upregulate the YadGH efflux/metabolic module without changing Yad fimbriae expression. Fimbriae are usually involved in initial colonization or surface adhesion (Korea et al., 2010). This functional decoupling gives bacteria finer control over stress responses. They can respond to microenvironmental pressures (like nitroaromatic exposure or acid stress) without the extra energy cost of expressing fimbriae.

Given this distinct regulatory autonomy, the structural organization of the YadGH components themselves warrants closer examination.

### 4.5 Structural architecture and functional implications of the YadGH efflux module

In *E. coli*, ABC family efflux pumps usually require multiple subunits to move substrates across the double membrane. The most typical examples are tripartite systems, such as MacAB-TolC (Huo et al., 2025) and the YbhG/YbhFRS-TolC system (Feng et al., 2020a). In these systems, a single polypeptide like MacB contains both the nucleotide-binding domain (NBD) and the transmembrane domain (TMD). They also require a periplasmic adaptor protein (like MacA) and an outer membrane channel (TolC) to work properly (Huo et al., 2025).

Recently, binary systems have also been found, such as the YddA-YddB pair (Feng et al., 2020b). Here, the inner membrane protein YddA interacts with the outer membrane beta-barrel protein YddB. This removes the need for a periplasmic adaptor. However, the exact details of how they transport substrates remain unclear.

The *yadGH* operon exhibits an architecture distinct from canonical tripartite (e.g., MacAB-TolC) or binary (e.g., YddA-YddB) systems. Its “split-subunit” organization—YadG providing the NBD and YadH the TMD—suggests that functional transport likely requires assembly into a heteromeric complex. Notably, the operon lacks genes encoding a periplasmic adaptor or an outer membrane channel. This raises the question of how substrates traverse the outer membrane; recruitment of TolC or other outer membrane factors remains to be demonstrated.

Consistent with this structural specialization, YadGH conferred only a modest increase in macrolide MICs compared to the major AcrAB-TolC pump. This limited impact suggests YadGH is not a broad-spectrum antibiotic exporter. Instead, it appears tailored for the transport of specific physiological substrates, potentially functioning in concert with other cellular pathways rather than as a standalone efflux route.

### 4.6 Implications and future directions

YadGH only confers low-level macrolide tolerance when the *acrB* gene is deleted, highlighting the strictly conditional nature of this efflux-mediated phenotype. This observation raises a key question: can YadGH aggravate drug resistance scenarios where the major AcrAB-TolC efflux machinery is impaired?

Although our work did not test clinical or veterinary isolates, the data suggest YadGH may act as a minor auxiliary contributing factor under such defective AcrAB backgrounds, rather than a dominant primary resistance determinant. Notably, the non-canonical weak promoter and distal MarA-dependent activation mode of *yadGH* (sections 4.1 and 4.2) demonstrate tight transcriptional control of this operon, meaning its phenotypic influence is only expected under narrow, specific environmental pressures.

Four complementary research directions are proposed to dissect the wider biological relevance of the MarA-YadGH regulatory axis. First, identifying endogenous physiological substrates of YadGH will clarify its core native function and the stimuli that trigger its natural expression. Untargeted metabolomics and LC-MS profiling can be applied to screen candidate substrates including bile salts, plant secondary metabolites, or central metabolic intermediates. Second, it remains unconfirmed whether YadGH operates as an independent inner membrane transporter or relies on outer membrane channels such as TolC for substrate extrusion. Co-immunoprecipitation combined with mass spectrometry can be used to characterize the full set of protein partners assembled with YadGH. Third, to probe any potential clinical or veterinary relevance of MarA-YadGH signaling, future work should profile *marA* and *yadGH* expression in extraintestinal pathogenic *E. coli* (ExPEC) isolates, and measure macrolide susceptibility in strains carrying loss-of-function acrB mutations. Lastly, *in vivo* colonization models (murine intestinal colonization or Galleria mellonella larval infection) can be deployed to evaluate whether YadGH improves bacterial fitness within host-associated microenvironments.

We stress that all translational interpretations drawn from the MarA-YadGH pathway remain purely speculative at this stage; dedicated, context-matched biological and clinical assays are required to validate these hypothesized real-world effects.

We also acknowledge that we did not conduct formal validation of primer amplification efficiencies in the present study. Melting curve analysis verified single specific amplicons for all primer sets, yet follow-up quantitative tests would be strengthened by experimentally measured amplification efficiencies to further validate expression results calculated via the 2^∧^−ΔΔCt method.

## 5 Conclusion

This study identifies the ABC efflux transporter YadGH as a transcriptional target of MarA in *E. coli* K-12. MarA binds to a degenerate marbox within the *yadGH* promoter, and MarA-dependent regulation of *yadGH* transcription was confirmed by qRT-PCR. Functionally, YadGH confers low-level resistance to macrolides (tylosin and erythromycin) only when the major efflux system AcrAB-TolC is compromised. In addition, YadGH affects biofilm formation and swarming motility, suggesting a broader physiological role beyond drug efflux. Together, these findings establish a molecular basis for MarA-dependent regulation of an auxiliary efflux pump and add to our understanding of how *E. coli* coordinates stress responses.

## Data Availability

The original contributions presented in the study are included in the article/supplementary material, further inquiries can be directed to the corresponding author.
